# Fast Delamination of Fuel Cell Catalyst-Coated Membranes Using High-Intensity Ultrasonication

**DOI:** 10.1016/j.ultsonch.2025.107330

**Published:** 2025-03-26

**Authors:** Tanongsak Yingnakorn, Ross Gordon, Daniel Marin Florido, Christopher E. Elgar, Ben Jacobson, Shida Li, Paul Prentice, Andrew P. Abbott, Jake M. Yang

**Affiliations:** a School of Chemistry, University of Leicester, Leicester LE1 7RH, United Kingdom; b School of Metallurgical Engineering, Suranaree University of Technology, Nakhon Ratchasima 30000, Thailand; c Johnson Matthey Technology Centre, Blounts Court Road, Sonning Common, RG4 9NH, United Kingdom; d James Watt School of Engineering, University of Glasgow, Glasgow G12 8QQ, United Kingdom

**Keywords:** Delamination, Fuel cell, High-intensity ultrasonication, Membrane, Catalyst, Insonation

## Abstract

This study demonstrates a rapid and facile method for separating the central membrane and catalyst-coated material from production scrap fuel cell catalyst-coated membranes (CCMs), facilitating a circular economy of technologically critical metals. A novel approach is presented using high-intensity ultrasonication with two distinct sonotrode configurations for rapid delamination at ambient temperature in water. This technique utilises cavitation, where high-frequency sound waves create, expand, and collapse microbubbles, generating high-speed jets, shockwaves, and acoustic streaming. This process effectively separates the membrane and catalyst while maintaining their overall integrity of the former. A cylindrical sonotrode (20 mm diameter) was used to optimise process parameters for smaller CCM samples to minimise time and energy consumption. To scale up the delamination process for industrial-size CCMs, a blade sonotrode (15 mm x 210 mm) was employed to enable a flow process for rapid and continuous delamination. Cavitation at the sonotrode-CCM interface was shown to facilitate the selective and rapid breakdown of the catalyst layers, enabling full delamination of the catalyst-loaded membrane within tens of seconds. This efficient and fast delamination approach offers a promising strategy for CCM recycling.

## Graphical abstract

**Figure:**
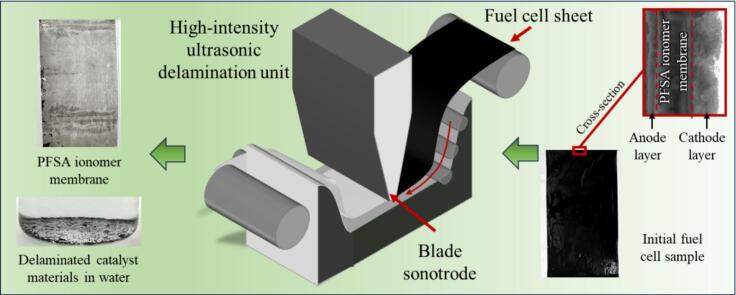

## 1 Introduction

Proton exchange membrane fuel cells (PEMFCs) are a prominent focus in renewable energy conversion technologies. They offer a method of greener, more cost-effective, and portable energy solutions [1], [2]. PEMFCs use hydrogen as fuel to generate electricity, with water as the sole by-product [3]. In PEMFCs, a central membrane (8–18 μm) is sandwiched between layers of cathode and anode catalyst material [4], [5], forming a composite structure known as a catalyst-coated membrane (CCM). The chemical composition of these catalyst layers can be tailored to achieve specific functionalities within the fuel cell. Platinum supported on carbon (Pt/C) is the predominant electrocatalyst for PEMFCs owing to its fast hydrogen oxidation kinetics [6]. To facilitate rapid proton transport to the catalyst’s active sites, perfluorosulfonic acid (PFSA) polymers are commonly employed as the ionomeric phase [7], [8]. A significant challenge lies in the recovery of these precious catalytic materials as fuel cells at end-of-life (typically 10–15 years) or from production scrap material [2], [9], [10]. Therefore, the ability to recover and reuse precious metal catalysts (such as platinum and iridium), as well as expensive PFSA-based ionomer membranes, becomes critical. This underscores the need for sustainable recovery and reuse of materials for the long-term sustainability of PEMFC technology.

Current recycling technologies for PEMFCs prioritise the recovery of platinum group metal (PGM) catalysts due to their higher market value [11], [12], [13], [14]. However, the ionomer, another essential PEMFC component, is often disregarded despite its significant intrinsic value. Additionally, due to their wide range of industrial and consumer product uses, per- and polyfluoroalkyl substances (PFAS), of which PFSA ionomers are a small subgroup, are increasingly detected in the environment. The majority of PFAS are environmentally persistent (half-life estimates for soil and water range from years to decades) [15], [16], and certain PFAS have been shown to bioaccumulate along food chains and to be harmful to human health [17]. On the basis of these concerns, for critical technologies that currently rely on PFAS, it is essential that losses to the environment, particularly at end-of-life, are minimised, ideally through recovery and recycling [3], [18], [19], [20]. Recognising this discrepancy, Johnson Matthey developed their innovative HyRefine^TM^ technology to achieve co-recovery of valuable PGMs and ionomers for reuse in new CCMs [21]. This chemical approach enhances CCM recycling efficiency and sustainability compared to conventional PGM refining. Separation of the ionomer membrane is a critical step in the recycling process from CCMs, allowing for the independent recovery of both the membrane and the metal components. A common method utilises alcohol-water mixtures, such as isopropyl alcohol, 1-butanol, or 2-butanol, heated to an elevated temperature of 100–200 °C. These conditions are believed to disrupt the bonds between the fluorocarbon-based ionomer and the catalyst particles, causing the membrane to disperse in the solvent solution, facilitating separation [22]. Xu et al. report a method for Nafion membrane recycling via immersion in boiling isopropanol (20 min) for swelling, followed by mechanical removal of catalyst residues. The pre-treated membranes were then boiled in 3–5 % H_2_O_2_ solution (1 h) for complete decolourisation [23]. Alternatively, supercritical water (350–450 °C, 200–400 bar, 1–10 h) offers selective separation of fluorine-containing components (ionomers, etc.) from precious metals in spent fuel cells [24].

Ultrasonication, using high-frequency sound waves (20–100 kHz) [25], has emerged as a versatile tool across various scientific disciplines [26]. It has been used in numerous applications, including the dispersion of nanoemulsions and nanocomposite materials [27], [28], [29], separating water from crude oil [30], generating Hg(OH)_2_ from Hg^0^(l) [31], as well as the crucial task of recycling and extracting valuable metals [32], [33]. Applications for this technology span a wide range, including pharmaceutical and food sciences, biodiesel production, water treatment, cleaning, and removal of fine particles [34], [35], [36]. Notably, ultrasonic soundwaves introduce forced convection through three primary mechanisms: cavitation collapse, microjet formation, and acoustic streaming [37]. High-power ultrasound also enables rapid mass transport within a system, removing passivating surface layers during electrochemical stripping of metals to expose fresh reaction sites [38], [39], and even facilitating the delamination of multi-layered materials [40]. Recent advancements include the development of an ultrasonic blade designed explicitly for the delamination of lithium-ion battery anode and cathode sheets [41], [42]. The current research explores the potential of using a high-power ultrasound technique as a fast, simple, and ambient temperature process for the efficient separation of CCMs. By strategically positioning the CCM directly under the sonotrode, we aim to achieve delamination in under ten seconds. This rapid process facilitates the separation of the catalyst layers from the central membrane, enabling the recovery of both components. The concentrated ultrasonic cavitation and acoustic pressure generated at the sonotrode interface are expected to be sufficient to break the bond between the catalyst layers and the membrane. We further optimise this method for large-scale production scrap membrane processing by changing the sonotrode configuration from a small cylinder to a novel blade configuration to facilitate continuous processing. This efficient and rapid delamination approach offers a promising strategy for CCM recycling with significant scale-up potential.

## 2 Experimental section

### 2.1 Materials

Production scrap fuel cell CCMs were provided by Johnson Matthey. These CCMs comprised a polymer membrane with a PFSA ionomer membrane coated on each side with a catalyst layer. The catalyst layers were composed of combinations of platinum on carbon (Pt/C) or a combination of Pt/C and iridium-based metal oxide (IrMO_x_) nanoparticles. Ethanol (≥99.8 %, VWR) was used as the solvent for soaking the fuel cell CCMs before delamination.

### 2.2 Delamination using the ultrasonic systems

**Fig. 1:**
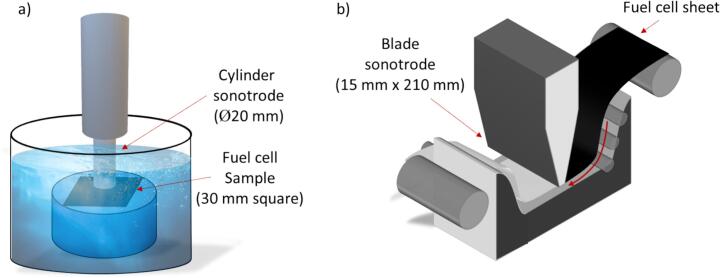
schematic images of ultrasonic delamination a) with a cylindrical sonotrode, and b) with a custom-built blade sonotrode. Notice that the commercially available cylindrical sonotrode configuration is set up to delaminate CCMs in batches, whereas the blade sonotrode is optimised for continuous processing by incorporation of roller blades to facilitate continuous sample feed.

A commercial ultrasonic delamination system (Branson Sonics, 1.25DCXa20-V, Danbury, CT, USA) was employed to separate the CCM of the fuel cell. The CCM sample was first cut into a 30 mm x 30 mm square (45.3 ± 1.7 mg). Subsequently, the sample was submerged in ethanol for a predetermined duration, ranging from 5 to 60 s. After ethanol treatment, the CCM sample was placed onto the smooth surface of a stainless-steel rod and then positioned within a beaker filled with deionised water (200 ml). The delamination process utilised a vertical configuration with a cylinder sonotrode (horn tip, diameter of 20 mm) placed at a fixed distance directly above the CCM sample, as illustrated in Fig. 1**a**. The ultrasonic system operated at a frequency of 20 kHz and offered adjustable power ranging from 125 W to 1250 W, translating to a variable power intensity of c.a. 40 W cm^−2^ to 400 W cm^−2^.

The work herein optimised parameters for the ultrasonic delamination process. The parameters explored included ultrasonic power, sonication time, and the perpendicular distance of the sonotrode-to-sample distance. The ultrasonic power was varied at four levels: 10 % (ca. 40 W cm^−2^), 20 % (ca. 80 W cm^−2^), 30 % (ca. 120 W cm^−2^), and 40 % (ca. 160 W cm^−2^) of the system's maximum capacity. Each power level was combined with three sonication times: 1 s, 2 s, and 5 s. Additionally, the effect of sonotrode-to-sample distance was evaluated at two settings: 2.5 mm and 5 mm.

The optimal parameters identified during ultrasonic delamination with a cylindrical horn were subsequently employed for a scaled-up delamination process. This involved a rapid and continuous delamination of a large fuel cell membrane using a high-power ultrasonic unit. This custom-designed unit incorporated a blade sonotrode with a rectangular cross-section measuring 15 mm x 210 mm, as shown in Fig. 1**b**. The blade sonotrode could deliver a maximum power intensity of 70 W cm^−2^. A sonication bath was constructed using a stainless-steel tray positioned within a tank containing approximately 6 L of deionised water. The sonotrode was placed 5 mm above the tray. A large, industrial-scale CCM sample measuring 80 mm x 170 mm was fed through the blade sonotrode. Prior to sonication, samples were immersed in ethanol for 30 s to undergo a pretreatment process. Plastic sheets were affixed to both ends of the CCM sample to maintain sample integrity and to facilitate continuous feeding during the sonication process. The prepared sample was passed through the sonication bath at a constant velocity of about 1 cm s^−1^ with the blade sonotrode operating at 40 W cm^−2^ of power intensity. Catalyst-coated materials were dispersed within the aqueous medium (DI water). Following complete delamination of both sample surfaces, the delaminated membrane and suspended catalyst materials in water were retrieved and characterised using SEM and EDS.

To investigate the impact of fuel cell delamination without the support of a stainless-steel rod, a 30x50 mm^2^ sample was secured using a custom-designed clamping apparatus (Fig. S1). Ultrasonic delamination was induced under optimal conditions using a cylindrical sonotrode, with a power intensity of 40 W/cm^2^ and a sonotrode-to-sample distance of 5 mm. Additionally, to establish a baseline for delamination efficiency, CCM sample sections (30 mm x 30 mm) were initially immersed in ethanol for 30 s, followed by ultrasonic treatment in deionised (DI) water for 30 min using a Fisherbrand® FB15055 ultrasonic bath (200–240 V, 50/60 Hz, 550 W). The DI water was then replaced, and sonication was continued for an additional 10 min. These samples, assumed to be fully delaminated (as illustrated in Supplementary Fig. S2), exhibited a mean mass reduction of 36.18 ± 1.67 % relative to their initial mass. This mass variation served as a reference standard for the comparative gravimetric determination of delamination efficiency achieved by the blade sonotrode method.

### 2.3 Other instrumentation

High-speed imaging (Fastcam SA-Z 2100 K, Photron, Bucks UK) was employed to capture the cavitation activity and streaming phenomena associated with fuel cell delamination. Images were acquired at a frame rate of 20,000 frames per second using a macro lens (Milvus 100 mm f/2 M, Zeiss, Oberkocken Germany). Synchronous 10 ns collimated laser pulses (632 nm, red) (CAVILUX Smart, Cavitar, Finland) were utilised for illumination, enabling shadow graphic imaging and providing precise temporal resolution.

Scanning electron microscopy (SEM) was performed using a Zeiss Gemini 360 FEGSEM to characterise the morphology and elemental composition of the samples, as well as any particles remaining on the membrane substrate after delamination. The SEM was operated in in-lens mode with an accelerating voltage of 1 kV and a spot size of 5 nm for imaging. Energy-dispersive X-ray spectroscopy (EDS) analysis was performed using an Oxford Instruments Ultim Extreme windowless detector (Oxford Instruments, Abingdon, UK) at an accelerating voltage of 5–15 kV, controlled using Aztec software.

## 3 Results and Discussion

The effectiveness and scalability of ultrasound-facilitated delamination of production scrap fuel cell CCM are studied herein. Firstly, the delamination parameters were optimised using a commercially available cylindrical sonotrode with a horn diameter of 20 mm. Subsequently, the optimised parameters obtained from the first part of this study are transferred to a custom-built blade sonotrode (15 mm x 210 mm) equipped with rollers to facilitate a continuous delamination of CCM materials to demonstrate scalability.

The production scrap fuel cell CCM was used for delamination studies. The CCM comprised three distinct layers with a total thickness of approximately 35 µm: a Pt/C cathode layer (14.53 µm ± 1.37 µm), a central PFSA ionomer membrane layer (14.57 µm ± 1.24 µm), and Pt/C, IrMO_x_ anode layer (4.50 µm ± 0.75 µm). Square-shaped samples (30 mm x 30 mm) were prepared from the CCM to facilitate the optimisation of the delamination process. A high-intensity ultrasonic delamination technique employing a cylindrical sonotrode submerged in a water bath was utilized at ambient temperature to assess the influence of various parameters on delamination efficiency. Before sonication, the samples underwent a pretreatment stage involving immersion in ethanol for 30 s. As detailed in our previous work, this pretreatment soaking step was implemented to modify, swell and soften the rigidity of the particle-loaded Nafion film, significantly reducing the sonication time required to fully separate central membranes from catalyst active materials [43].

The parameters, including ultrasonic horn power, the spacing between the sonotrode and the sample, and the sonication time, were optimised to enhance operational efficiency and minimise energy consumption. Experiments were conducted with a sonotrode with a diameter of 20 mm at a fixed sonotrode-to-sample distance. Ultrasonic horn power output was varied from 10 % to 40 % of the maximum power setting in 10 % increments, and sonication time of 1 s, 2 s, and 5 s were evaluated. As expected, the delamination area increased when sonication power increased (Fig. S3). Interestingly, as can be seen in Fig. S3, delamination of the CCM with the anode side (a thin layer composed of Pt/C and IrMO_x_ particles) facing the sonotrode results in a more transparent central membrane as compared to the case if the cathode side (a thicker layer composed of Pt/C particles) was facing the sonotrode. In all cases, under excessively high power and long sonication times (e.g., 40 % power and 5 s at a 5 mm distance), irreversible damage (ripping) to the middle membrane layer was seen, and this potentially hinders the reusability of the recovered membrane. Similarly, reducing the sonotrode-to-sample distance to 2.5 mm also caused similar damage (as shown in Fig. S4). This observation aligns with the established principle that sound wave pressure and intensity increase at distances closer to the sonotrode surface [44], [45]. Notably, a setting of 10 % power and 1 s of sonication of both sides of the membrane at a 5 mm distance away from the sonotrode was sufficient for effective delamination (Fig. S5).

**Fig. 2:**
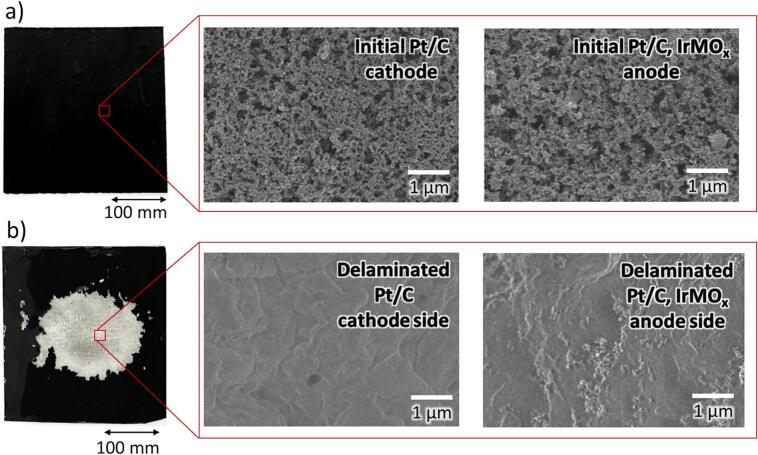
Shows SEM images of the a) initial fuel cell at the Pt/C cathode side and the Pt/C, IrMO_x_ anode side and the b) clean area of delaminated membrane surfaces at the Pt/C cathode and the Pt/C, IrMO_x_ anode side (40 W cm^−^^2^, 5 mm sonotrode-to-sample distance, and 1 s of sonication for each side of the sample). Full EDS analysis of the anode and cathode elements is shown in **Fig. S6**.

Fig. 2 shows SEM images of the CCM sample before and after two-sided delamination using the above-optimised parameters. In this case, both sides of the CCM were subject to 1 s of sonication in deionised water at 10 % power output (ca. 40 W cm^−2^) and a 5 mm sonotrode-to-sample distance. As can be seen in Fig. 2**b**, both layers of catalyst-active materials were successfully removed, revealing the quasi-transparent PFSA membrane. SEM-EDS analysis was used to characterise the material before and after sonication. Fig. 2**a** shows the morphologies of the Pt/C cathode and Pt/C, IrMO_x_ anode layers before delamination. The elemental identity of ‘M’ is commercially sensitive, and its identity is therefore not revealed as it is unimportant for this work. The delaminated surfaces of membrane surfaces after the sonication treatment are presented in Fig. 2**b**. Post-delamination EDS analysis of the recovered membrane revealed only carbon, oxygen, fluorine, and sulphur, with no detectable traces of catalyst materials. The distribution of elements from EDS studies is tabulated in Table S1.

**Fig. 3:**
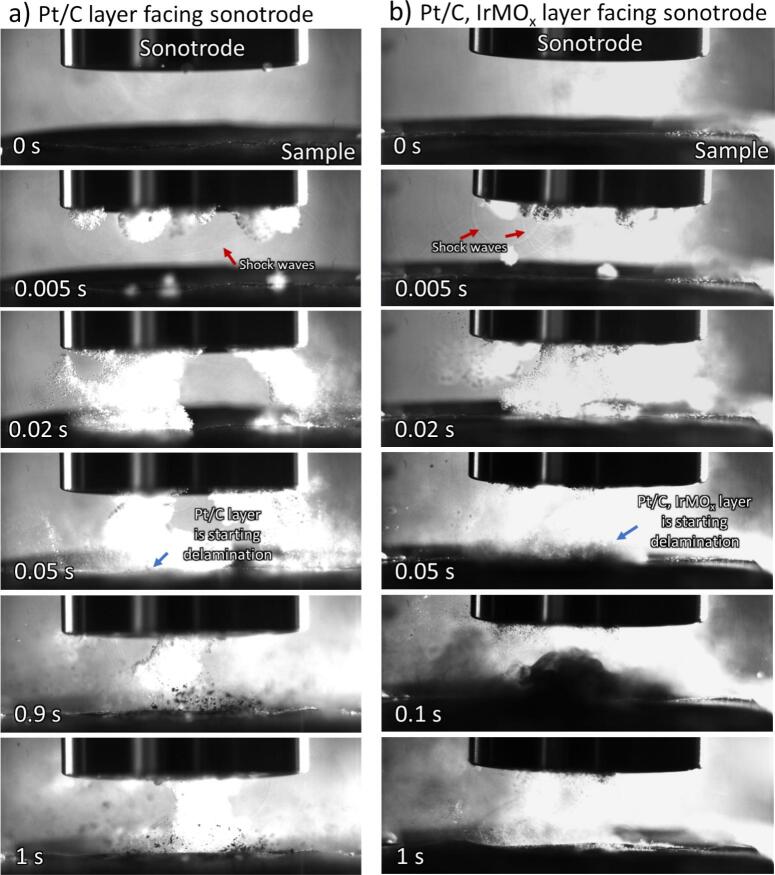
Images showing snapshots taken from a high-speed camera (20 k frames per s) during ultrasonic delamination of the fuel cell sample in water: (a) Pt/C cathode side facing sonotrode; (b) Pt/C, IrMO_x_ anode side facing sonotrode. The sonotrode was 20 mm in diameter, with 40 W cm^−2^ power intensity, at 5 mm from the substrate.

High-speed imaging was employed to investigate the effects of cavitation of bubbles during the sonication of a fuel cell sample. Many studies have utilised this technique to directly observe cavitation activity, using high frame rates to capture the intricate dynamics of bubble formation and collapse near surfaces so as to provide new physical insights into the delamination processes [39], [46], [47]. Fig. 3 presents the delamination behaviour of pristine, untreated fuel cell samples with the Pt/C (Fig. 3**a**) and, separately, Pt/C, IrMO_x_ side (Fig. 3**b**) facing the sonotrode. The sonotrode was operated at a power intensity of 40 W cm^–2^ at a fixed distance of 5 mm from the CCM substrate suspended in water. Sonication initiation at t = 0 s generates stochastic bubble clouds at the surface of the cylindrical ultrasonic horn tip. After ca. 5 ms, smaller, variable-sized bubble clusters near the horn tip surface are observed. During sonication, the violent collapse of bubble clouds and cavitation implosions within the cavitation region between the sonotrode and the sample resulted in the generation of shockwaves and micro-jets to enhance mass transport to and from the surface of the sample at a short distance of 5 mm from the sonohorn [48]. Concomitantly, acoustic streaming, characterised by fluid circulation in the vicinity of bubbles, forces fluid toward the sample surface (observed at 20 ms) [49]. After 50 ms, cavitation led to the delamination of catalyst-active materials from the CCM substrate [41], [50]. Interestingly, the delamination behaviour of the Pt/C cathode side and Pt/C, IrMO_x_ anode side facing the sonotrode exhibited significant differences, as revealed by the high-speed camera. While the delamination of the Pt/C cathode side resulted in small but sizeable flakes (evidenced at 0.9 s), the delamination of the Pt/C, IrMO_x_ anode side emanates a ‘cloud’ of finer particles (at 0.1 s). Moreover, the delamination of the Pt/C, IrMO_x_ anode side appeared to be more facile and resulted in a larger delamination area. These disparities in detachment mechanisms may be attributed to compositional differences in materials and layer thickness differences between the cathode and anode catalyst layers. Full length delamination videos can be found online as part of the Supplementary Information Videos S1 and S2.

**Fig. 4:**
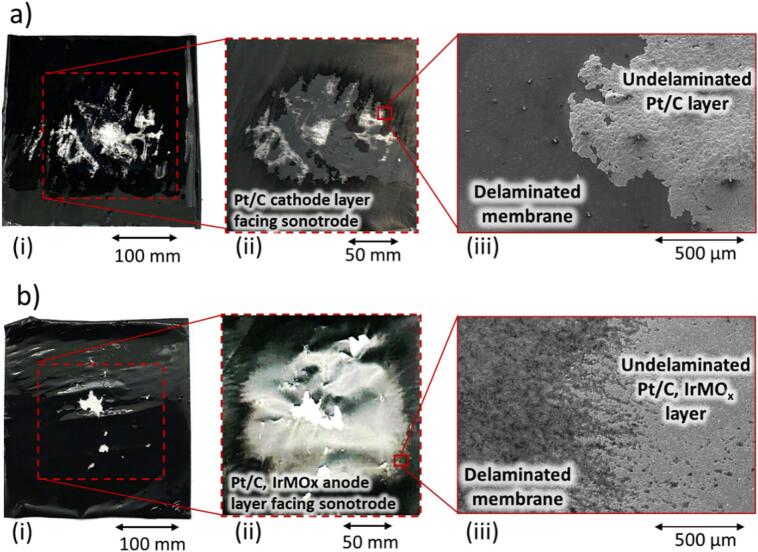
Delamination of fuel cell CCM induced by ultrasonic cylinder sonotrode. Images of (a) the Pt/C cathode side and (b) the Pt/C, IrMO_x_ anode side of a CCM sample after ultrasonic delamination in water (10 % power, 1-second sonication time, 5 mm sonotrode-sample distance). (i) Initial sample, (ii) sample after drying (image adjusted for brightness and contrast), and (iii) corresponding SEM image of the delamination interface. For the detailed elemental analysis of regions indicated in (iii), refer to **Fig. S7**.

Fig. 4**a(i)** shows the optical image of the delaminated CCM with the Pt/C cathode facing the sonotrode and shown in Fig. 4**b(i)** is a separate experiment with the anode side (Pt/C, IrMO_x_) facing the sonotrode. To enhance the visibility, Fig. 4a**(ii)** and Fig. 4**b(ii)** are replicas of Fig. 4**a(i)** and Fig. 4**a(ii)**, respectively, but the brightness and contrast have been enhanced to better display the extent of delamination on the membrane surface. While successful delamination occurred on the Pt/C cathode side, the opposite side of the membrane exhibited significant residual material (not shown). Regardless of which side of the CCM is facing the sonotrode, there is a consistent radial delamination pattern revealing the part of the membrane that is closest to the sonotrode. This is consistent with the above physical insights obtained from high-speed imaging, where self-organised conical cavitation fields directly under a sonotrode operating at low frequencies and high power are evidenced [51], [52]. While a clean area is observed on the Pt/C, IrMO_x_ anode side after delamination, the opposite side of the membrane (not shown) still retains coated material. Note that the weakly coated materials may detach from the central membrane during the sample drying. Furthermore, the interfacial regions between delaminated and intact catalyst layers, SEM images shown in Fig. 4**a(iii)** and **4b(iii)**, exhibit distinct morphological characteristics. On the Pt/C cathode side, the catalyst coating appears to detach in cohesive failure, with undelaminated particles remaining on the surface in a flake-like structure. In contrast, the Pt/C-IrMO_x_ anode interface reveals a more adhesive failure mode, where small, micron-sized agglomerates of individual particles are observed to remain on the surface. These findings are consistent with the high-speed imaging, which revealed flakes of material emanating from the Pt/C surface and clouds of fine particles emanating from the Pt/C-IrMO_x_ surface.

Additionally, the findings suggest the possibility of achieving one-sided delamination (without flipping the sample for further sonication) when sonicating commences from the Pt/C, IrMO_x_ anode layer facing the sonotrode. This effect is particularly evident at higher power levels and extended sonication times, such as 20–30 % of power for 5 s, resulting in cleaned areas on both sides, as shown in Fig. S3. This is supported by the observation of cleaned areas on both sides of the membrane after delamination due to the propagation of ultrasonic waves through the membrane, resulting in delamination of both sides (see the shock waves across the sample in Fig. S8). This finding suggests a viable strategy for laboratory-scale separation of cathode and anode catalyst materials, particularly when the respective layer characteristics are known. By designing a specialized containment vessel that prevents the mixing of the delaminated materials, while leveraging the membrane as an inherent barrier (conceptualized in Supplementary Fig. S9), selective catalyst recovery may be facilitated. However, initiating sonication from the Pt/C side resulted in an unclean membrane surface, potentially due to the thicker and different material properties of the Pt/C layer compared to the Pt/C, IrMO_x_ anode layer, which could hinder effective ultrasonic wave transmission and delamination. Consequently, due to the practical challenge of consistently identifying the anode layer from the cathode, which both appear black to the naked eye, a two-sided delamination approach is adopted to ensure clean membrane surfaces on both sides of the CCM sheet.

Ethanol pre-soaking is critical to achieve successful delamination. As anticipated, sonication without this pretreatment resulted in incomplete separation, as evidenced by the substantial presence of undelaminated material in the sample (Fig. S10). This observation reinforces the hypothesis that the pre-soaking stage is essential for modifying the material properties of the CCM, facilitating effective delamination and ultimately leading to the acquisition of fully separated membranes [43]. Furthermore, while the investigation explored the influence of pre-soaking time on delamination efficiency, the findings revealed no statistically significant variations in results between 10 and 60 s. This suggests that the soaking time within this range may be a minor factor for delamination success. However, to maintain consistency throughout the study, a constant soaking time of 30 s will be adopted for further experiments.

**Fig. 5:**
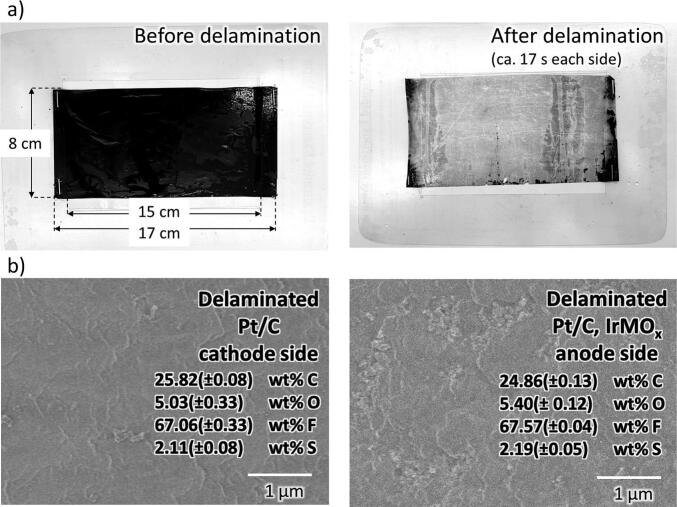
Characterisation of fuel cell CCM samples before and after the delamination process. (a) Comparative images of CCM samples before and after delamination. (b) SEM images of the membrane surface after delamination at the Pt/C cathode side and Pt/C, IrMO_x_ anode side. Delamination conditions: blade sonotrode, 40 W cm^−2^ power intensity, 1 cm s^−1^ viscosity feeding speed, ambient temperature, 5 mm sonotrode-to-sample distance.

Building upon the optimal delamination conditions identified with the cylinder sonotrode (30 s ethanol pre-soaking, 40 W cm^−2^ power intensity, and 5 mm sonotrode-to-sample distance), these parameters were then applied to the custom-built blade sonotrode. A fuel cell CCM sample of 80 mm x 170 mm dimensions was fed under the blade sonotrode at a speed of approximately 1 cm s^−1^. This allows continuous delamination of large CCM which is otherwise not possible using the smaller cylindrical sonotrode. The successful delamination experiment is visually documented in Supplementary Video S3. The resulting effects of ultrasonic delamination on both the anode and cathode sides of the CCM can be visualised in the SEM images of the membrane after the sonication treatment in Fig. 5. A comparative analysis of the initial and post-delamination stages of the scrap fuel cell CCM (Fig. 5**a**) reveals a successful removal of the catalyst-coated layers from the central membrane polymer through ultrasonic sonication. Subsequent SEM-EDS analysis of the delaminated membrane surfaces detected negligible catalyst residues (Fig. 5**b**), mirroring the findings obtained with the cylinder sonotrode. Visual inspection of the delaminated sample (Fig. 5**a**) reveals minor residual staining, which is attributed, in part, to optical artifacts such as air entrapment shadows and variations in surface wetting against the white background (see Supplementary Fig. S11 for illustrative examples). Despite these visual imperfections, the delaminated membrane exhibited a high degree of clarity and transparency. Quantitatively, the delamination efficiency of the PFSA ionomer membrane, achieved through the blade sonotrode method, was determined to be 99.28 (±0.92)%. This value was derived from a gravimetric analysis, comparing the residual mass of the delaminated membrane samples using the sonotrode blade technique (after removal of overlap regions with the plastic sheet) to the mass of fully delaminated reference samples, as detailed in the experimental section and illustrated in Supplementary Fig. S2. Note that the ultrasonic delamination process experienced a temperature increase from ca. 25 °C to 35 °C within 5 min of sonication. This thermal escalation suggests that implementing a cooling system may be useful to ensure the process's stability and reliability during continuous operation. While the blade sonication method employed in this study demonstrated efficacy in delaminating intact sheet or roll-format samples, however, there might be other challenges associated with shredded materials with irregular or fragmented geometries. A potential alternative, continuous delamination within a high-intensity ultrasonic bath or tank [53], could be explored for processing irregular or fragmented materials, such as production waste, disassembled components, or materials with diverse geometries and conditions. The current investigation serves as a proof-of-concept, validating the potential of high-intensity sonication for complete sheet separation in proton exchange membrane recycling and recovery.

After ultrasound delaminations, the catalyst materials, which are insoluble in water, remain in the aqueous solution (Fig. S12), which requires further separation. This could be achievable through conventional techniques such as filtration, sedimentation, centrifugation, or evaporation [54], [55], [56], [57], [58]. Alternatively, if the mixture of particles has a disparity in hydrophilicity, it could be separated by froth flotation or other means [59]. Depending on the state of the material before delamination, subsequent processing may involve reprocessing platinum group metals (PGMs) back into catalyse precursor materials, which is typically facilitated by hydrometallurgical methods [14], [60], [61], [62], [63] (see Table S3 for examples of the processes). While the delamination process effectively isolates the PFSA membrane, its direct reuse in catalyst-coated membrane (CCM) fabrication for fuel cells requires further investigation. Observed membrane deformation and potential damage from high-power sonication suggest that direct CCM refabrication may be challenging as the membrane itself may need to be first recast [23], [64], [65], [66], [67]. Alternatively, the recovered ionomer can be a constituent of the catalyst layer, incorporated into catalyst ink through dispersion with catalyst particles [8], [68]. However, the approach presented in this work offers significant advantages over conventional recycling methods. Compared to pyrometallurgical techniques, which operate at high temperatures (up to 1100 °C) to concentrate PGMs [69], [70], our method operates at room temperature, conserves valuable membrane material, minimizes noxious emissions, and lowers energy consumption. Furthermore, it addresses limitations associated with direct membrane dissolution using solvents like ethanol, methanol, DMF, DMSO, and DMAc at elevated temperatures (80–240 °C). These conventional dissolution methods often encounter issues such as incomplete dissolution, filtration challenges, prolonged processing times (hours to days), and the potential requirement for high-pressure reactors [23], [64], [71], [72]. The proposed delamination process provides a more efficient and sustainable alternative, circumventing these challenges while enabling effective material recovery.

## 4 Conclusion

This study demonstrated the potential of high-intensity ultrasonic delamination for separating catalyst layers from the PFSA ionomer membrane in fuel cell CCMs. The investigation identified vital parameters influencing the delamination process, including ultrasonic power, sonication time, and sonotrode-to-sample distance. The optimised protocol achieved clean delamination with minimal damage through a fast process. Firstly, a 30-second ethanol pretreatment effectively modified the rigidity of the catalyst-loaded membrane, significantly impacting the subsequent delamination step. Secondly, sonication was performed using a conservative 10 % ultrasonic horn power (40 W cm^−2^) for a brief duration (1 s per side) at a controlled sonotrode-sample distance (5 mm) at ambient temperature in water.

The efficacy of these optimised parameters was further validated using a blade sonotrode, demonstrating its capability of continuous delamination of large pieces of production scrap CCMs at a rate of 80 mm^2^ s^−1^ with minimal ionomer membrane damage. As the first generation of fuel cells is now approaching their end of life, the insights presented herein provide a green, rapid, highly scalable, low-cost close-loop separation and recycling of valuable components like the PFSA ionomer membrane and catalyst materials.

## CRediT authorship contribution statement

**Tanongsak Yingnakorn:** Writing – review & editing, Writing – original draft, Visualization, Investigation, Conceptualization. **Ross Gordon:** Writing – review & editing, Supervision, Conceptualization. **Daniel Marin Florido:** Writing – review & editing, Supervision, Conceptualization. **Christopher E. Elgar:** Writing – review & editing, Supervision. **Ben Jacobson:** Investigation. **Shida Li:** Investigation. **Paul Prentice:** Writing – review & editing, Supervision. **Andrew P. Abbott:** Writing – review & editing, Supervision, Funding acquisition, Conceptualization. **Jake M. Yang:** Writing – review & editing, Supervision, Conceptualization.

## Declaration of competing interest

The authors declare that they have no known competing financial interests or personal relationships that could have appeared to influence the work reported in this paper.
