# Supplementary material for: Fast Delamination of Fuel Cell Catalyst-Coated Membranes Using High-Intensity Ultrasonication
Source: Ultrason Sonochem. 2025 Mar 26;116:107330. doi: 10.1016/j.ultsonch.2025.107330 (PMC11995761; doi:10.1016/j.ultsonch.2025.107330)
Supplement: Supplementary Data 1 [file mmc1.docx]

**Fast Delamination of Fuel Cell Catalyst-Coated Membranes Using High-Intensity Ultrasonication**

Tanongsak Yingnakorn^1,2^, Ross Gordon^3^, Daniel Marin Florido^3^, Christopher E. Elgar^1^, Ben Jacobson^4^, Shida Li^4^, Paul Prentice^4^, Andrew P. Abbott^1^, Jake M. Yang*^*^*^1^

*^1^ School of Chemistry, University of Leicester, Leicester, LE1 7RH, United Kingdom*

*^2^ School of Metallurgical Engineering, Suranaree University of Technology, Nakhon Ratchasima 30000, Thailand*

*^3^ Johnson Matthey Technology Centre, Blounts Court Road, Sonning Common, RG4 9NH, United Kingdom*

^4^ *James Watt School of Engineering, University of Glasgow, Glasgow G12 8QQ, United Kingdom*

*^*^jake.yang@leicester.ac.uk*

**Supplementary information**

**
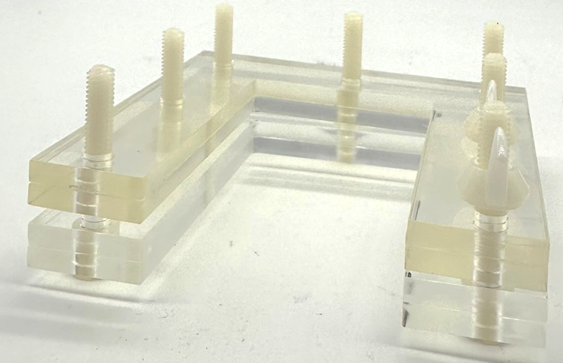
**

**Figure S1** Photos of a custom-designed device for clamping of the sample for investigation of the delamination during sonication and transmitted shockwaves passing through the sample.

**
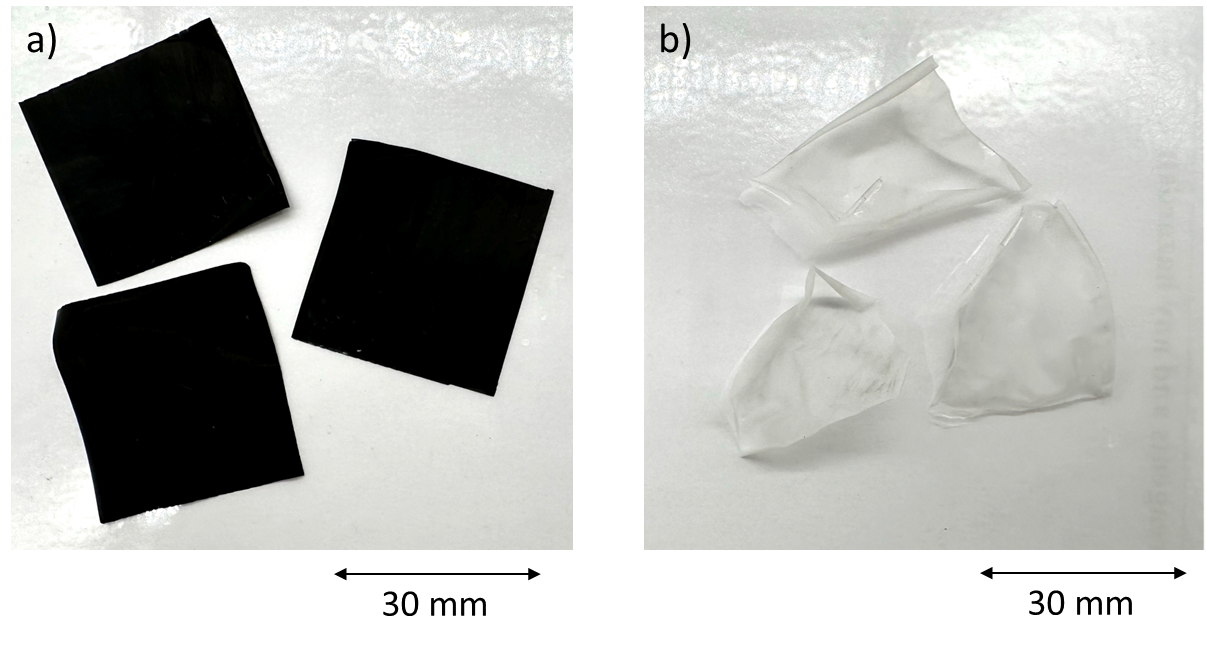
**

**Figure S2** Photos of a) initial fuel cell CCM and b) fully delaminated samples that served as a reference point for comparative analysis of the delamination efficiency experimental conditions in this study (immersed in ethanol for 30 seconds, followed by sonication in water for 30 minutes using an ultrasonic bath, then replaced with fresh water, and continued sonication for an additional 10 minutes). The mass variations of the fuel cell CCM reference samples were 36.18 (±1.67) %, relative to their initial mass prior to delamination.

**
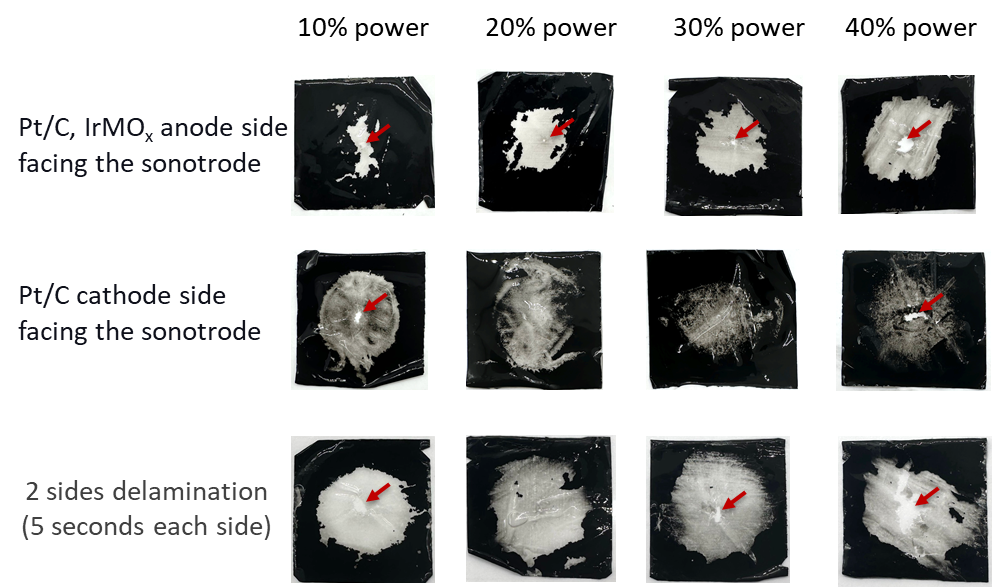
**

**Figure S3:** The fuel cell samples (30 mm x 30 mm) delaminated in water under various sonication conditions. The sample was subjected to 5 seconds of sonication at different power levels while facing the sonotrode (the red arrows show the broken areas). A constant sonotrode-to-sample distance of 5 mm was maintained.

**
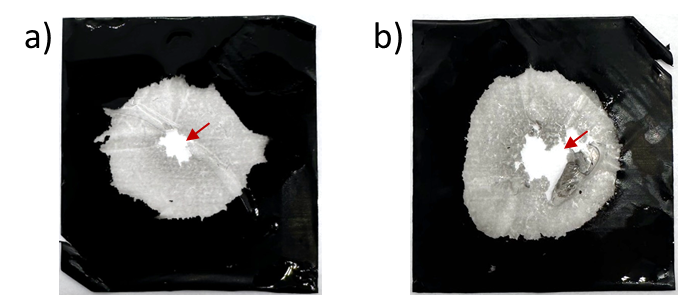
**

**Figure S4** The fuel cell samples (30 mm x 30 mm) delaminated in water with a) 10% and b) 20% of power at fixed 1-second sonication and sonotrode-to-sample distance of 2.5 mm (the red arrows show the broken areas).


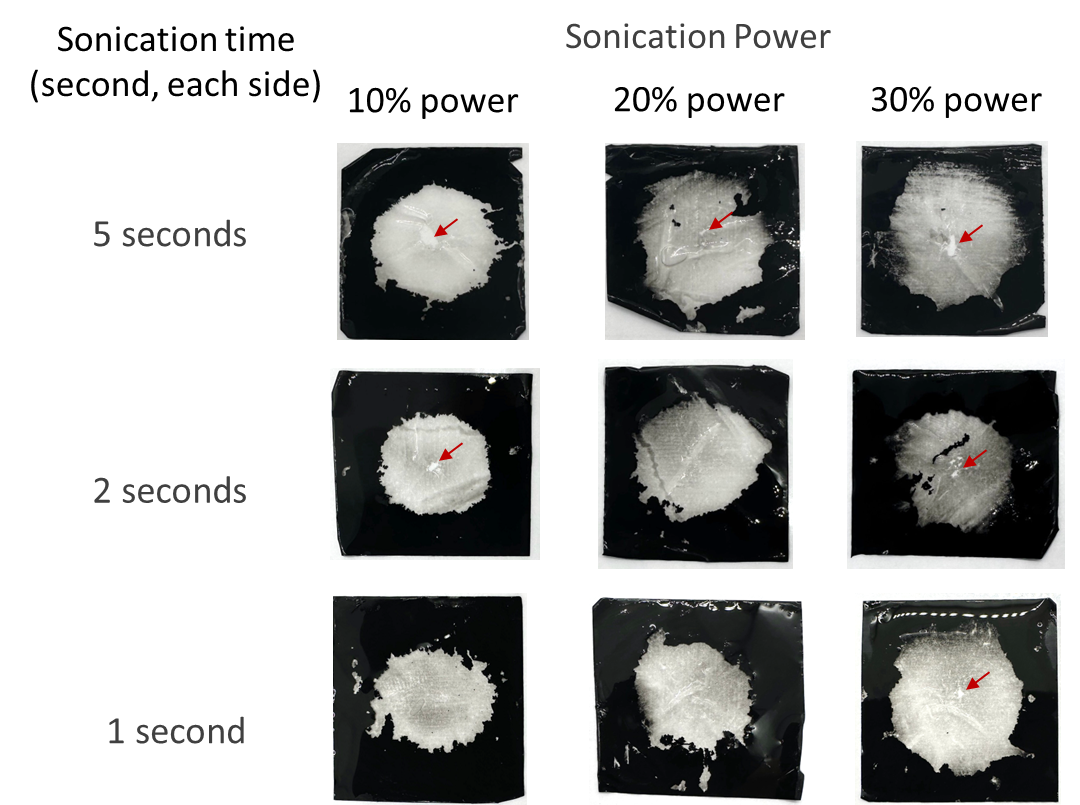


**Figure S5** The 2-sides delaminated fuel cell samples (30 mm x 30 mm) in water at different sonication times (on each side) and ultrasonic powers (the red arrows show the broken areas).


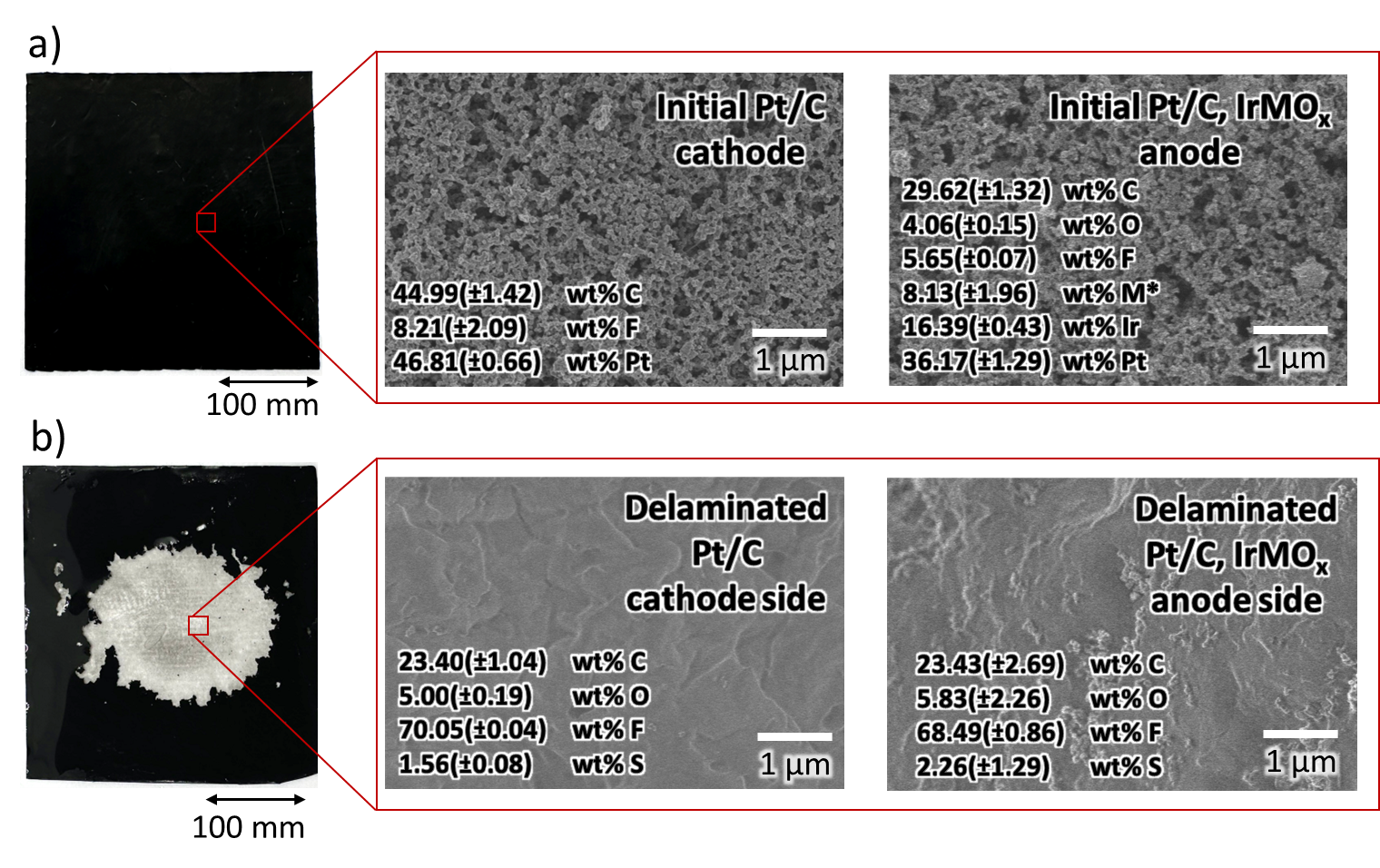


**Figure S6** shows SEM images and EDS results of the a) initial fuel cell at the Pt/C cathode side and the Pt/C, IrMO_x_ anode side and the b) clean area of delaminated membrane surfaces at the Pt/C cathode and the Pt/C, IrMO_x_ anode side (40 W cm^-^², 5 mm sonotrode-to-sample distance, and 1 second of insonication for each side of the sample). * The element 'M' in the image has been removed from EDS analysis due to commercial sensitivity. The identity of M is unimportant to this work.


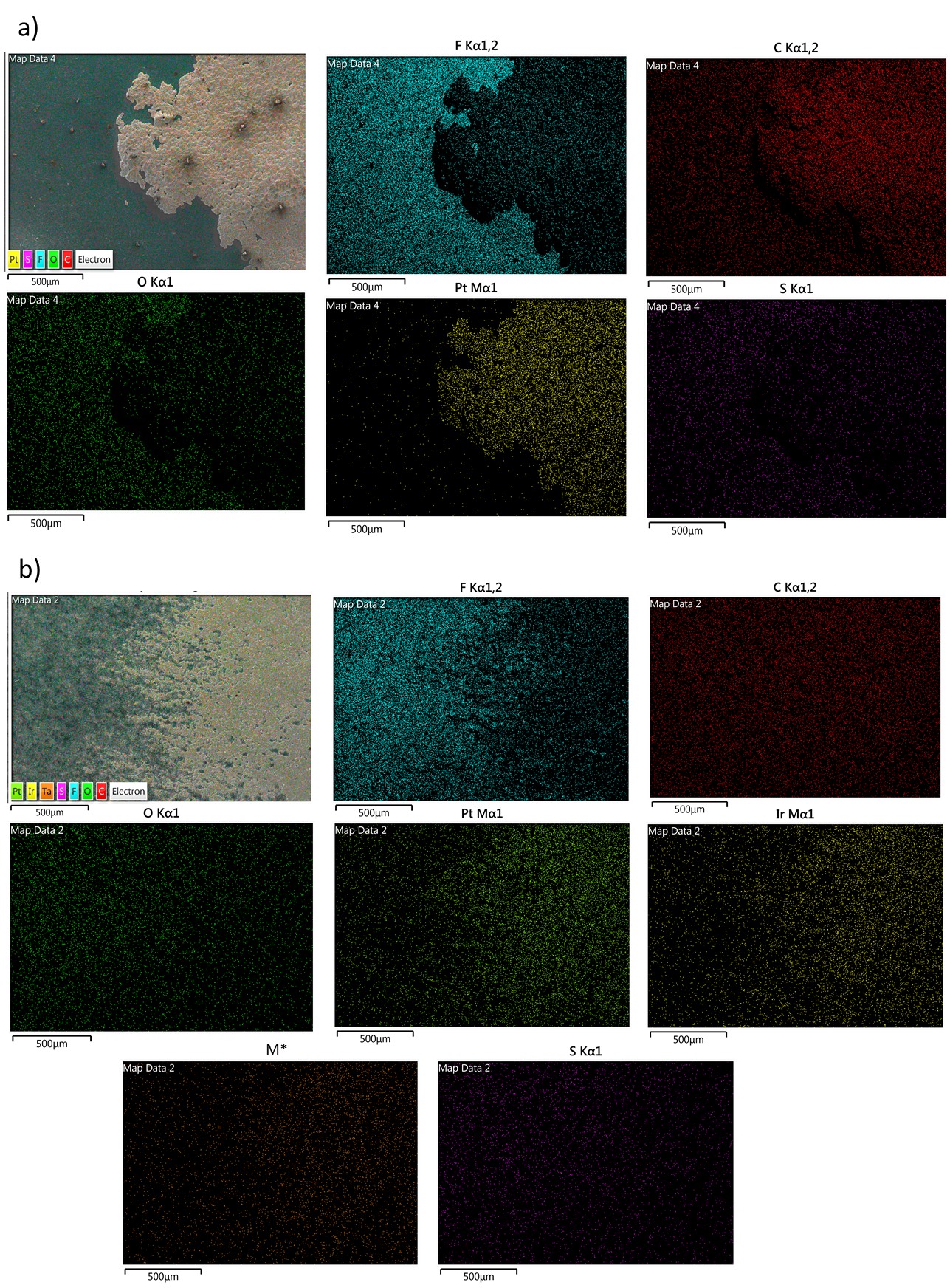


**Figure S7** EDS element mapping of the delaminated fuel cell sample in water (10% power, 1-second sonication time, 5 mm sonotrode-to-sample distance) at the edge of delaminated and under laminated areas; a) Pt/C, IrMO_x_ anode side facing the sonotrode. b) Pt/C cathode side facing the sonotrode. * The element 'M' in the image has been removed from EDS analysis due to commercial sensitivity. The identity of M is unimportant to this work.

**
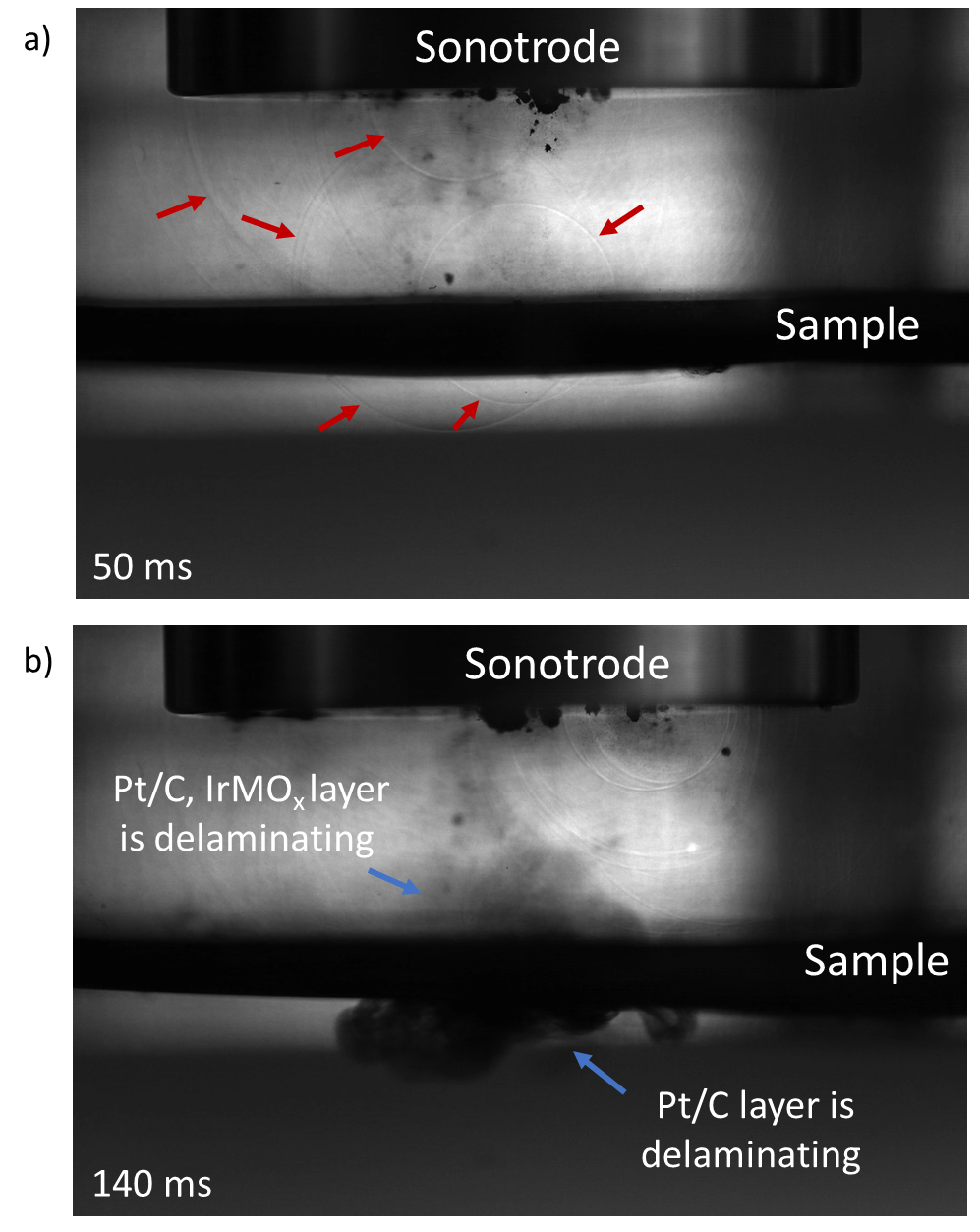
**

**Figure** **S8** High-speed imaging of a) shockwaves generated by collapsing bubbles near the cylindrical sonotrode, with the transmitted shockwaves passing through the sample at 50 ms (indicated by the red arrow), and b) delaminating of catalyst-coated materials from both layers at 140 ms. The sample was secured using a custom-designed clamping and sonicated at 40 W/cm² of power intensity and 5 mm of sonotrode-to-sample distance.


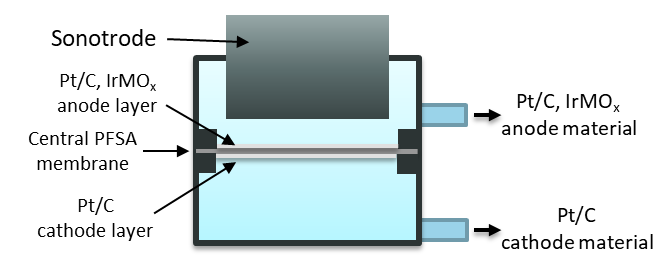


**Figure** **S9** Conceptual design for anode and cathode catalyst material isolation during delamination by sonication.

**
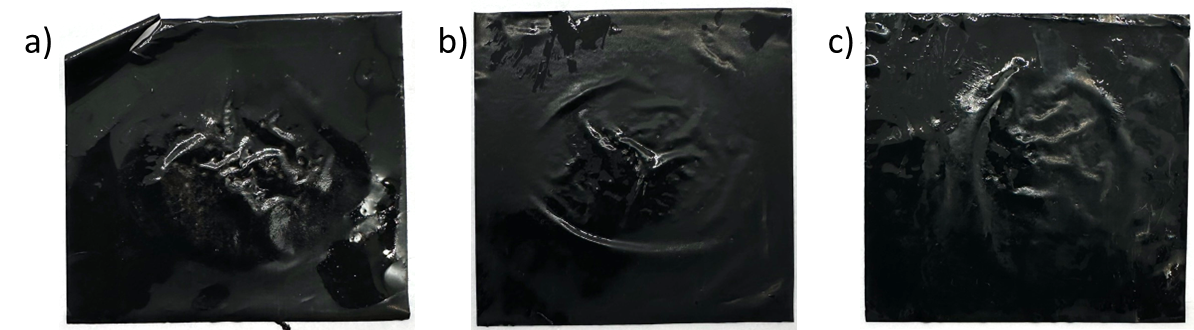
**

**Figure S10** Delamination results of fuel cell samples (30 mm x 30 mm) sonicated 2-sides (1 second for each side) in water at a) 10% power, b) 20%, and c) 30% power without ethanol soaking.

**
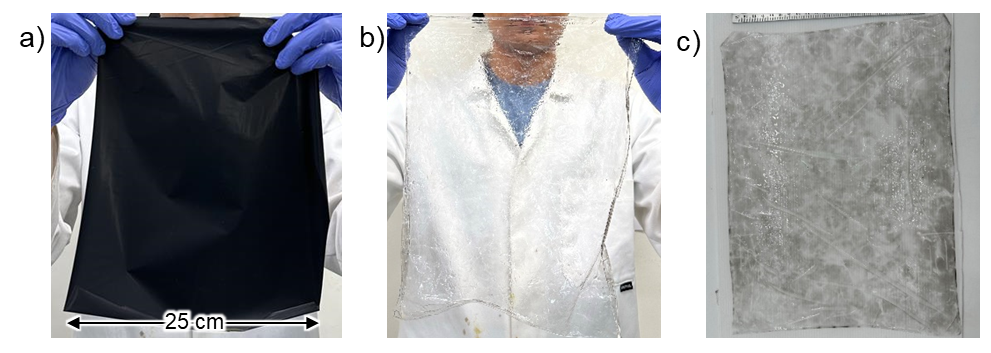
**

**Figure S11** Visual comparison of delaminated fuel cell samples with and without a white background: a) the initial fuel cell sample, b) the delaminated fuel cell sample (after ethanol soaking and sonicating) that was taken without a white background board, and c) the delaminated fuel cell sample that was taken a picture from the top view on the white background. Note: These images illustrate the visual differences arising from varied image capture techniques and are derived from samples delaminated using a 1-minute ethanol soak followed by a 1-hour low-power ultrasonic bath, a similar method to our previous study**[1]** . This sample is distinct from the delaminated sample by the blade sonotrode method employed in the current investigation.

**
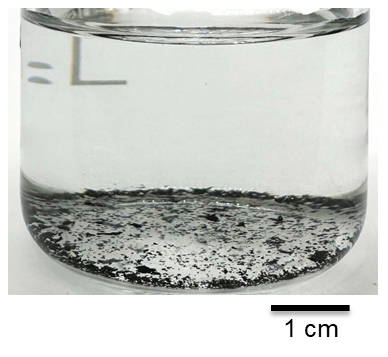
**

**Figure S12** Delaminated catalyst materials in water media after sonication under the condition of 40 W cm^-^² power intensity and 5 mm sonotrode-to-sample distance.

**Table S1** Chemical composition of the surfaces of initial CCM and the delaminated membrane after sonication with the cylinder sonotrode (40 W cm^-^², 5 mm sonotrode-to-sample distance, and 1 second of sonication for each side of the sample).

| **Initial CCM** | | | |
| --- | --- | --- | --- |
| **Layer** | **Element** | **Weight%** | **Atomic%** |
| Pt/C cathode side | C | 44.99($\pm$1.42) | 84.79($\pm$2.45) |
|  | F | 8.21($\pm$2.09) | 9.80($\pm$2.53) |
|  | Pt | 46.81($\pm$0.66) | 5.43($\pm$0.06) |
| Pt/C, IrMO_x_ anode side | C | 29.62($\pm$1.32) | 73.98($\pm$1.29) |
|  | O | 4.06($\pm$0.15) | 7.62($\pm$0.49) |
|  | F | 5.65($\pm$0.07) | 8.93($\pm$0.34) |
|  | M* | 8.13($\pm$1.96) | 1.36($\pm$0.36) |
|  | Ir | 16.39($\pm$0.43) | 2.56($\pm$0.14) |
|  | Pt | 36.17($\pm$1.29) | 5.57($\pm$0.05) |
| **Delaminated CCM surfaces** | | | |
| Pt/C cathode side | C | 26.20($\pm$1.10) | 35.97($\pm$1.35) |
|  | O | 1.96($\pm$0.12) | 2.02($\pm$0.13) |
|  | F | 70.87($\pm$1.17) | 61.52($\pm$1.31) |
|  | S | 1.00($\pm$0.19) | 0.49($\pm$0.06) |
| Pt/C, IrMO_x_ anode side | C | 26.33($\pm$1.41) | 36.09($\pm$1.76) |
|  | O | 2.94($\pm$1.83) | 3.02($\pm$1.92) |
|  | F | 69.56($\pm$0.66) | 60.29($\pm$0.28) |
|  | S | 1.19($\pm$0.21) | 0.61($\pm$0.11) |

* The element 'M' in the image has been removed from EDS analysis due to commercial sensitivity. The identity of M is unimportant to this work.

**Table S2** Chemical composition of the surfaces of the delaminated membrane after sonication with the blade sonotrode (40 W cm^-^² power intensity, 1 cm s^-1^ viscosity feeding speed, 5 mm sonotrode-to-sample distance).

| **Layer** | **Element** | **Weight%** | **Atomic%** |
| --- | --- | --- | --- |
| Pt/C cathode side | C | 26.20($\pm$1.10) | 35.97($\pm$1.35) |
|  | O | 1.96($\pm$0.12) | 2.02($\pm$0.13) |
|  | F | 70.87($\pm$1.17) | 61.52($\pm$1.31) |
|  | S | 1.00($\pm$0.19) | 0.49($\pm$0.06) |
| Pt/C, IrMO_x_ anode side | C | 26.33($\pm$1.41) | 36.09($\pm$1.76) |
|  | O | 2.94($\pm$1.83) | 3.02($\pm$1.92) |
|  | F | 69.56($\pm$0.66) | 60.29($\pm$0.28) |
|  | S | 1.19($\pm$0.21) | 0.61($\pm$0.11) |

**Table S3** Hydrometallurgical PEMs recovery processes. Reproduced with permission from [1]

| Initial material | Leaching  method | Solvent extraction | | Precipitation  (% efficiency) | Recovery product | Ref. |
| --- | --- | --- | --- | --- | --- | --- |
|  |  | Extraction | Stripping |  |  |  |
| Spent catalyst (Pt dust) | Aqua regia for 1.5 h at 109 ºC (S/L = 0.1) | - | - | NH_4_Cl to precipitate (NH_4_)_2_PtCl_6_ | Ignition to get Pt powder 97.9 purity.  (98% recovery) | [2] |
|  |  | 10% trioctylamine (TOA) in kerosine | NH_4_OH/ NH_4_Cl | - | Ignition to get Pt powder 99.9%purity. |  |
| PEMFC (calcination at 600 ºC for 6 h to eliminate the carbon) | HNO_3_ at 65 ºC added HCl and heated at 110 ºC to vaporise fully | - | - | The solid H_2_[PtCl_6_] is dissolved in water again at a pH value between 3.0 and 4.0 by adding 0.5 mol ml^-1^ NaOH. HCOOH is then added to the solution to reduce the platinum. | H_2_[PtC_l6_] solution | [3] |
| CCMs | H_2_SO_4_ at 150 ºC for 72 h. adjusted to 4–6 with 2 M NaOH solution (filter to separate sediment) | - | - | Pt(SO_4_)_2_ in solution is reduced by adding Cu powder | Pt powder | [4] |
| MEA | HCl, H_2_O_2_ (S/L = 0.00236g/g) | C_8_H_18_O, Cyanex 923 | NaOH solution | HCl, NH_4_Cl, H_2_O | (NH_4_)_2_PtCl_6_ | [5] |
| MEA from PEMFC | 12.5 M HCl, 3vol% H_2_O_2_ at 25 °C for 5 h | [P_44414_]Cl | 1M HCl precipitation | - | [P44414]_2_[PtCl_6_], Co in aqueous phase, | [6] |
|  |  | [C_14_ pyr][NTf_2_] | - | - | Pt(IV) in ionic liquid, Co(II) in the aqueous phase |  |
| PEMFC, MEAs | HCl, 3 vol% of H_2_O_2_ (95.0% of Pt, 99.0% of Co leaching efficiency) for 5h 25 ºC (S/L = 4x4 cm^2^/200ml) | 15 vol% of Cyanex 923 extractant diluted in octanol solvent (99.4% Pt efficiency) | NaOH solution (90.1% Pt efficiency) | - | Co oxide (84.0% overall efficiency), PtCl_6_^-2^(85.0% overall efficiency) in alkaline solution | [7] |
|  |  | Lewatit-MP62 resin (R/A = 20 g L^−1^) (99.0% Pt efficiency) | NaOH solution (82.7% Pt efficiency) | - | Co^2+^ solution (47.0% overall efficiency), PtCl_6_^2-^ (78.0% overall efficiency) in alkaline solution |  |
|  | 1 M HCl, H_2_O_2_ for 2 h (∼99%) | - | - | 2M NH_4_Cl (∼98%) | (NH_4_)_2_PtCl_6_ (∼97%) | [8] |
| MEA | 1 M HCl, 3% 1.5 vol % H_2_O_2_ (50 mL/MEA) for 24 h (98%) | - | - | 5 M NaOH (pH=13) | Ru precipitate, Pt solution | [9] |
| MEAs | 1 M HCl 80 ºC for 48 h | - | - | C_2_H_6_O_2_ (5 mg/ml Pt in EG), NaOH (1:10) reflux at 180 ◦C for 3h, | Pt colloid in EG | [10] |
| MEA (50% IPA solution and sonication for 30 min at 70 C) | 5 M HCl 10% H_2_O_2_ at 70 ºC for 120 min (90%) | - | - | - | H_2_PtCl_6_ aqueous solution | [11] |
| PtCl_6_^2−^ and IrCl_6_^2−^ solution | - | [Bmim]PF_6_ or 4-(bromomethyl) benzoate and EBTOA]Br, [EBTPEA]Br, or [EBTPA]Brin a 1:3 M ratio | 0.25 M NH_2_OH·HCl solution to extract Pt(IV)  , then 30%(w/w) H_2_O_2_ and [hydrochloric acid](https://www.sciencedirect.com/topics/chemical-engineering/hydrochloric-acid) was added to extract Ir(IV) | - | Pt(IV) and Ir(IV) | [12] |
| simulated the PEMFC electrodes ((40 wt.% Pt [nanoparticles](https://www.sciencedirect.com/topics/earth-and-planetary-sciences/nanoparticle)   on Vulcan XC72)) | H_2_O_2_/HCl (91% efficiency) and HNO_3_/HCl 93% efficiency) for 24 h at 25 °C | Cyanex 923/Octanol | NaOH  solution | NH_4_Cl, HCl | (NH_4_)_2_PtCl_6_ | [13] |
|  |  | Lewatit-MP-62 resin | NaOH  solution | NH_4_Cl | (NH_4_)_2_PtCl_6_ |  |

**References**

[1] T. Yingnakorn *et al.*, "Catalyst coated membranes for fuel cell and water electrolyser delamination induced by organic solution soaking and water ultrasonication," *RSC Sustainability,* 10.1039/D4SU00795F 2025, doi: <https://doi.org/10.1039/D4SU00795F>.

[2] M. A. Barakat and M. H. H. Mahmoud, "Recovery of platinum from spent catalyst," *Hydrometallurgy,* vol. 72, no. 3-4, pp. 179-184, 2004, doi: <https://doi.org/10.1016/S0304-386X(03)00141-5>.

[3] L. Zeng *et al.*, "Double recovery and regeneration of Pt/C catalysts: Both platinum from the spent proton exchange membrane fuel cell stacks and carbon from the pomelo peel," *Electrochimica Acta,* vol. Volume 428, 2022, doi: <https://doi.org/10.1016/j.electacta.2022.140918>.

[4] F. Xu, S. Mu, and M. Pan, "Recycling of membrane electrode assembly of PEMFC by acid processing," *International Journal of Hydrogen Energy,* vol. 35, no. 7, pp. 2976-2979, 2010, doi: <https://doi.org/10.1016/j.ijhydene.2009.05.087>.

[5] L. Duclos, M. Lupsea, G. Mandil, L. Svecova, P.-X. Thivel, and V. Laforest, "Environmental assessment of proton exchange membrane fuel cell platinum catalyst recycling," *Journal of Cleaner Production,* vol. 142, pp. 2618-2628, 2017/01/20/ 2017, doi: <https://doi.org/10.1016/j.jclepro.2016.10.197>.

[6] M. Gras *et al.*, "A Comparison of Cobalt and Platinum Extraction in Hydrophobic and Hydrophilic Ionic Liquids: Implication for Proton Exchange Membrane Fuel Cell Recycling," *ACS Sustainable Chem. Eng.,* vol. 8, no. 42, pp. 15865–15874, 2020, doi: <https://doi.org/10.1021/acssuschemeng.0c04263>.

[7] L. Duclos *et al.*, "Closing the loop: life cycle assessment and optimization of a PEMFC platinum-based catalyst recycling process," *Green Chem. ,,* vol. 22, pp. 1919-1933, 2020, doi: <https://doi.org/10.1039/C9GC03630J>.

[8] R. Sharma, i. J. Larsen, L. C. Larsen, A. Bogø, L. Grahl-Madsen, and S. M. Andersen, "A green adaptation of the critical resource of platinum group metals (PGMs) from spent autocatalysts to electrocatalysts for renewable power conversion," *Sustainable Energy Fuels,* vol. 6, pp. 5177-5186, 2022, doi: <https://doi.org/10.1039/D2SE00733A>.

[9] R. Sharma, S. Gyergyek, P. B. Lund, and S. M. Andersen, "Recovery of Pt and Ru from Spent Low-Temperature Polymer Electrolyte Membrane Fuel Cell Electrodes and Recycling of Pt by Direct Redeposition of the Dissolved Precursor on Carbon," *ACS Appl. Energy Mater.,* vol. 4, no. 7, pp. 6842–6852, 2021, doi: <https://doi.org/10.1021/acsaem.1c00964>.

[10] M. Chourashiya, R. Sharma, S. Gyergyek, and S. M. Andersen, "Gram-size Pt/C catalyst synthesized using Pt compound directly recovered from an end-of-life PEM fuel cell stack," *Materials Chemistry and Physics,* 2022, doi: <https://doi.org/10.1016/j.matchemphys.2021.125439>.

[11] W.-S. Chen, W.-S. Liu, and W.-C. Chen, "Leaching Efficiency and Kinetics of Platinum from Spent Proton Exchange Membrane Fuel Cells by H_2_O_2_/HCl," *Metals   2023,* vol. 13, no. 6, p. 1006, 2023, doi: <https://doi.org/10.3390/met13061006>.

[12] M. Fan *et al.*, "Separation and recovery of iridium(IV) from simulated secondary resource leachate by extraction - electrodeposition," *Separation and Purification Technology,* vol. 289, p. 120765, 2022, doi: <https://doi.org/10.1016/j.seppur.2022.120765>.

[13] L. Duclos, L. Svecova, V. Laforest, G. Mandil, and P. X. Thivel, "Process development and optimization for platinum recovery from PEM fuel cell catalyst," *Hydrometallurgy,* vol. 160, pp. 79-89, 2016, doi: <https://doi.org/10.1016/j.hydromet.2015.12.013>.
